# Advancing Precision Agriculture and Forestry: Multi-Source Spectral Sensing, Feature Fusion, and Machine Learning

**DOI:** 10.3390/plants15010003

**Published:** 2025-12-19

**Authors:** Youzhen Xiang, Zhiying Liu

**Affiliations:** 1 Key Laboratory of Agricultural Soil and Water Engineering in Arid and Semiarid Areas of Ministry of Education, Northwest A&F University, Yangling 712100, China; 2024055914@nwsuaf.edu.cn; 2 Xinjiang Research Institute of Agriculture in Arid Areas, Urumqi 830091, China

Building on the thematic foundation established in the first volume of this Special Issue—namely, leveraging spectral technologies (from proximal sensing to unmanned aerial vehicle (UAV) and satellite platforms) to advance precision agriculture and forestry—this second edition further consolidates methodological progress and expands the breadth of applications. Collectively, the papers emphasize the increasingly tight coupling among spectral information extraction, machine learning/deep learning modeling, and management-oriented decision support for sustainable agro-ecosystems.

A prominent thread in this edition is the diagnosis of plant stress and physiological status using both conventional reflectance indices and simplified imaging surrogates. Two contributions systematically demonstrate how red–green–blue (RGB)-based indices (low-cost, operationally scalable) can track stress-relevant spectral/physiological signals. One identifies drought-sensitive broadband normalized-difference reflectance configurations, and introduces an RGB-implementable normalized red–green index for drought detection in wheat and pea [1], while another establishes quantitative links between RGB indices and normalized difference vegetation index (NDVI)/photochemical reflectance index (PRI) as well as photosystem II (PSII) photochemical efficiency (Fv/Fm) under drought and salinization [2]. Complementing these empirical pathways, a mechanistic/analytical study explains why PRI and its modifications can vary beyond purely photosynthetic regulation, highlighting the roles of leaf-surface/mesophyll light scattering and anthocyanin dynamics (including drought-induced changes) [3].

A second core theme concerns crop water status characterization and water management, spanning canopy thermal responses, evapotranspiration estimation, soil moisture monitoring, and irrigation decision-making. Infrared imaging-based canopy temperature (CT) is evaluated as an integrative physiological indicator to differentiate wheat genotypes and relate thermal behavior to yield formation and grain quality traits [4]. At the field-to-regional scale, a satellite-assisted food and agriculture organization (FAO)-56 crop-coefficient approach replaces standard Kc values with vegetation-index-derived coefficients to derive actual evapotranspiration (ETa) for pumpkin, demonstrating a practical path for integrating Sentinel-2 dynamics and in situ soil observations in data-limited irrigation settings [5]. For root-zone water status monitoring, UAV multispectral texture information is advanced beyond conventional descriptors through novel three-dimensional texture indices and feature-fusion modeling to estimate soil moisture content across depths in arid croplands [6]. Finally, the management loop is closed by a data-driven irrigation prediction/intervention framework for *Panax notoginseng* (Burkill) F. H. Chen, integrating deep-learning time-series architectures to provide early irrigation warnings and reduce irrigation frequency while maintaining/improving growth outcomes [7].

In parallel, multiple papers deepen nutrient and quality diagnostics, demonstrating how hyperspectral/vegetation-index design and machine learning can support fertilization strategies and quality-oriented management. One study develops fractional-order-differentially optimized hyperspectral indices and a random forest framework to estimate cotton leaf phosphorus content, providing a pathway for timely phosphorus status detection across cultivars and treatments [8]. For nitrogen, a winter-wheat investigation constructs traditional, two-dimensional, and three-dimensional optimal spectral indices, then shows that input-feature design and model selection (notably random forest [RF] with enriched multidimensional indices) substantially improve leaf nitrogen concentration estimation [9]. Extending “status-to-outcome” inference, rice quality indices (e.g., brown rice rate, taste value) and yield are predicted by integrating multi-source indicators across phenological periods, illustrating how multi-stage physiological/spectral information can strengthen prediction robustness [10]. Additionally, nitrogen form and rate are explicitly embedded into UAV-based yield prediction pipelines via leaf area index (LAI) estimation and red-edge index selection, enabling the identification of optimal fertilizer strategies under the tested conditions [11].

Methodologically, this edition shows particularly strong momentum in UAV-enabled canopy trait retrieval and yield-relevant biophysical inversion, especially through multi-source feature fusion and novel index construction. A winter oilseed rape study proposes three-dimensional texture indices generated via correlation-matrix-driven random combinations, and demonstrates that integrating vegetation indices, texture features, and 3D texture indices within machine learning (notably XGBoost) can substantially enhance LAI inversion accuracy [12]. Relatedly, a maize LAI framework systematically tests stacked/ensemble strategies (partial least squares regression [PLSR] combined with RF/support vector machine [SVM]/gradient boosting decision tree [GBDT]) and shows consistent gains from moving beyond VIs alone to incorporate texture features and texture indices, with improved performance under independent test conditions [13]. Together, these papers reinforce a broader inference; spatial–structural information (textures) and its multidimensional re-parameterization can be as decisive as spectral contrast, particularly when operational constraints favor multispectral UAV sensors over full hyperspectral systems.

Beyond croplands, this Special Issue also emphasizes plant/forest health protection and rapid detection technologies. One contribution evaluates ultra-high-resolution optical satellite imagery for pine wood nematode (PWN) damage identification using U-Net, highlighting the dominant importance of spatial resolution (and suggesting performance saturation near 0.3 m under the tested conditions) for the detection of infected trees across platforms [14]. At the proximal sensing end of the spectrum, a green-synthesized carbon-dot/MOF fluorescent probe (WA-CDs@MIL-101) is developed for the rapid detection of *Panax notoginseng* (Burkill) F. H. Chen leaf pathogen spores, supporting early warning of disease spread through sensitive fluorescence recovery responses [15].

Finally, the edition includes work that broadens the “spectral technique” framing toward integrated land-use and production systems. An agroforestry intercropping study (camphor forest–winter rapeseed) examines how slope and stand density jointly affect rapeseed growth and yield formation, identifying slope–density combinations that improve canopy/biomass traits while supporting winter land utilization on red-soil slopes [16]. Such system-level studies are important for ensuring that advances in sensing and inversion are embedded within realistic management contexts, where structural constraints and land-use objectives co-determine the value of information.

In summary, the second edition of this Special Issue advances both scientific understanding (e.g., mechanistic drivers of index behavior) and operational pathways (e.g., RGB proxies, UAV texture fusion, satellite-assisted ETa estimation, and decision-oriented irrigation prediction). As in the first volume, the papers collectively point toward an evolving paradigm where multi-source sensing, machine learning, and agronomic/forestry decision processes are increasingly integrated into a coherent monitoring-to-management chain.
